# Noncontact evaluation of autonomic nervous system activity during exercise by using video analysis

**DOI:** 10.3389/fdgth.2025.1536492

**Published:** 2025-03-05

**Authors:** Kanaru Fumimoto, Shima Okada, Ryohei Tsuji, Yusuke Sakaue, Naruhiro Shiozawa, Hieyong Jeong, Masaaki Makikawa

**Affiliations:** ^1^ Graduate School of Science and Engineering, Ritsumeikan University, Shiga, Japan; ^2^ Department of Robotics, Faculty of Science and Engineering, Ritsumeikan University, Shiga, Japan; ^3^ Glabal Innovation Research Organization, Ritsumeikan University, Shiga, Japan; ^4^ Faculty of Sport and Health Science, Ritsumeikan University, Shiga, Japan; ^5^ Department of Artificial Intelligence Convergence, Chonnam National University, Gwangju, Republic of Korea; ^6^ Research Organization of Science and Technology, Ritsumeikan University, Shiga, Japan

**Keywords:** autonomic nervous system activity, facial capillaries, complexion, circulatory system, RGB camera

## Abstract

**Introduction:**

Autonomic nervous system activity (ANSA) plays a crucial role in the physical condition experienced during exercise and prolonged physical activity. In other words, ANSA is related to exercise performance and physical condition. Therefore, it is important to continuously monitor ANSA during high-intensity and sustained exercise. To this end, an uncomplicated and noncontact measurement system is preferable. Hence, in this study, we propose a method for the noncontact measurement of capillary contraction and dilation state, representative of ANSA, using a common commercial camera.

**Methods:**

Specifically, we focused on alterations in the green value of facial video images, from which we derived the green-to-blue (G/B) ratio as an indicator of blood vessel dilation and contraction, and to facilitate assessment of their activity. We performed a validation experiment involving exercise tasks using an ergometer in 10 healthy adults (23 ± 1.6 years old). The G/B ratio shows the state of contraction and expansion of facial capillaries, and it was evaluated using heart rate as ground truth data of the fluctuation of autonomic nerve activity.

**Results:**

We observed an increase in heart rate with decreased G/B ratio during exercise in all subjects. Postexercise, the heart rate decreased but the G/B ratio increased.

**Discussion:**

During exercise, characterized by dominant sympathetic NSA, the G/B ratio decreased, and recovered after exercise when parasympathetic NSA was dominant. In this way, noncontact evaluation of ASNA was achieved by using the G/B ratio. In the future, this measurement system will be applied to functional tests for heat acclimation.

## 1 Introduction

Exercise is an important contributor of health. In recent years, the proliferation of home exercise equipment has made at-home workouts increasingly popular (1). However, engaging in self-directed exercise is associated with the risk of injuries and adverse physical effects (2). Autonomic nervous system activity (ANSA) and cardiovascular status are closely related to physical well-being, emphasizing the importance of monitoring these factors during exercise for safety (3, 4). Adjusting exercise intensity and duration based on an individual's daily cardiovascular status, which is influenced by ANSA, has been demonstrated to help managing physical well-being. When exercise intensity is appropriately calibrated, it can strengthen immune function and increase resistance to infections (5). However, excessive or overly strenuous exercise is counterproductive, highlighting the need to monitor one's physical state and adjust exercise intensity accordingly.

Furthermore, exercise training considering ANSA and cardiovascular status contributes to improved performance (6–8). Sustained optimal performance can extend an athlete's longevity, resulting in a healthier and more enduring exercise regimen. Further, maintaining normal cardiovascular activity and ANSA can reduce the risk of heat stroke (9).

In recent years, the heart rate measured by wearable devices has been mainly used to evaluate ANSA during exercise. However, wearing them during exercise often causes discomfort. In addition, if the core temperature exceeds 40°C, the risk of heat stroke is very high. Body temperature is an important index from the viewpoint of evaluating exercise performance, because elevated body temperature leads to muscle weakness and decreased reaction time. Contact thermometers are mainly used to measure body temperature, but they are complicated during exercise. Non-contact thermography has a long time constant for measurement and cannot be evaluated simultaneously with the heartbeat. In addition, since ANSA has both cardiovascular and thermoregulatory functions, it is necessary to evaluate the two functions.

We noted that the dilatation and contraction information of the peripheral blood vessels of the face contains this information (10). Peripheral blood vessels play a key role in regulating body temperature, enabling heat retention or dissipation through their dilation and contraction (11). Capillaries also fulfill a circulatory role by transporting blood and oxygen. In a healthy state, the deep body temperature increases during exercise, and skin vessels dilate (12). Therefore, a novel indicator of ANSA, particularly focusing on facial peripheral blood vessel dilation and contraction, is essential. In this study, we focused on changes in facial color that accompany the contraction and dilation of facial capillaries. When facial capillaries dilate, the increasing blood volume in the face results in a red face. In contrast, facial capillary constriction decreases blood volume, resulting in a pale face (13). This pallid appearance correlates with lowered blood pressure. These facial color characteristics are also used for medical diagnosis, underscoring the high significance of facial color information (14, 15).

Face images captured by common cameras contain facial color information. This information has been widely used in applications such as the acquisition of photoplethysmography signals and blood pressure estimation (16, 17). These applications are based on blood flow extraction and hemoglobin quantification to obtain data on blood vessels. Using green information from a standard red, blue, and green (RGB) camera, heart and respiratory rates can be inferred from the cheeks and lips (18). Further, an RGB camera can also be used together with radar to estimate heart and respiratory rates (19). Moreover, this information can be used to estimate emotions (20). Thus, green light information was used to estimate indices such as heart rate and blood pressure. However, because they are very sensitive to body movement, measuring them during exercise is difficult. These measurements are difficult to measure unless face movement is eliminated by placing the jaw on a table and fixing the jaw, for example. Therefore, a new evaluation method is needed to evaluate ANSA during exercise.

In this study, we aimed to develop a method to extract the facial color information associated with blood vessel dilation and contraction during exercise using images captured with a common web camera. Then, the verification experiment was carried out to evaluate the method.

## 2 Methods

### 2.1 Characteristics of color information in face images

Human skin is composed of three primary layers, starting from the surface, there are the epidermis, dermis, and subcutaneous tissue. The epidermis contains melanin pigment, and the dermis is lined with capillaries. Especially, the blood in the capillary contains hemoglobin. Skin color is mainly determined by two light-absorbing substances, melanin and hemoglobin (21). Arteries and veins in the subcutaneous tissue deliver nutrients to the skin and remove waste products, with a significant portion of this tissue comprising subcutaneous fat (22). There are no significant light-absorbing materials in this layer, and since minimal light reaches the subcutaneous tissue, we can assume that only total or wavelength-independent reflection occurs.

**Figure 1:**
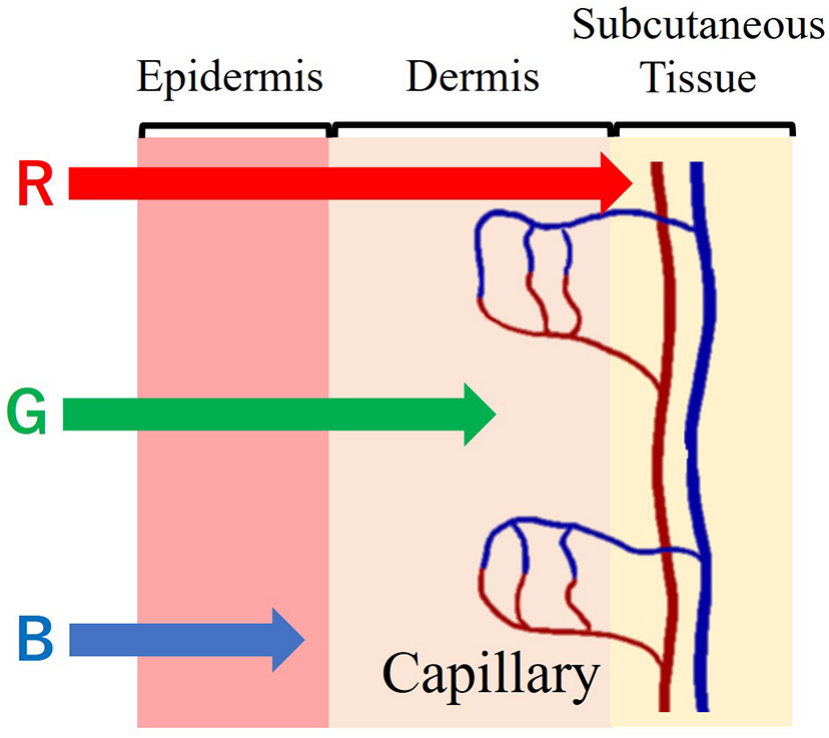
Cross-sectional structure of the skin and light wavelength penetration.

The penetration of light irradiated on the skin varies depending on its wavelength. Longer wavelength penetrates deeper into the skin. As such, short-wavelength blue light penetrates only the epidermis, while green light reaches the dermis, and longer-wavelength red light even reaches the subcutaneous tissue. Essentially, green and red light provide information about blood vessels. Figure 1 shows the correlation between the skin's cross-sectional structure and the penetration of light at different wavelengths.

**Figure 2:**
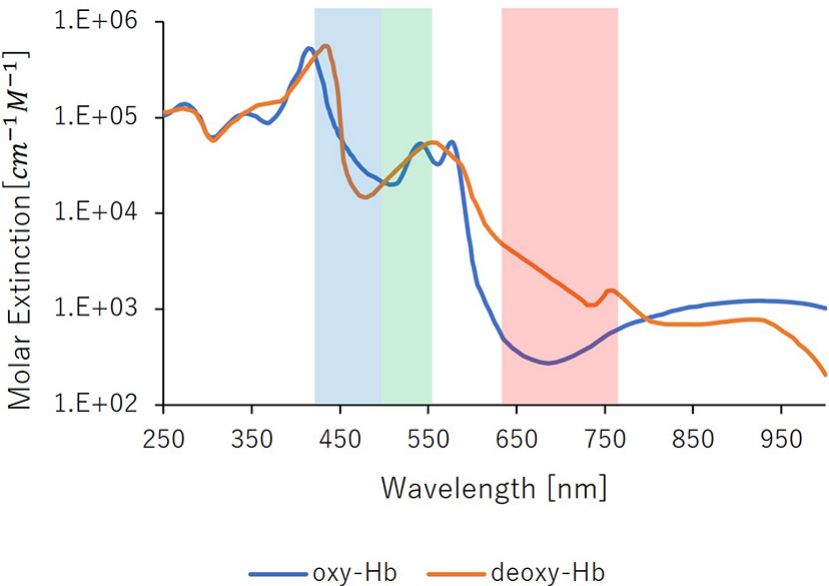
Absorption properties of hemoglobin.

Hemoglobin, a crucial component of blood, binds to oxygen in the lungs, where oxygen pressure is high, and releases it in tissues with lower oxygen pressure. Oxygen-bound hemoglobin, oxyhemoglobin (oxyHb), is bright red in arterial blood. Conversely, hemoglobin not bound to oxygen, deoxyhemoglobin (Hb), produces the dark red of venous blood. Figure 2 shows the absorbance characteristics of oxyhemoglobin and deoxyhemoglobin under light exposure (23). The absorbance properties of the two differ. However, oxyhemoglobin constitutes approximately 85% of the hemoglobin in blood vessels. Therefore, the absorbance properties of the fluid in entire vessels are similar to those of oxyhemoglobin. Figure 2 depicts the relationship between the light wavelength and the absorbance coefficient of hemoglobin. The larger the vertical axis of the molecular absorption coefficient, easier light is absorbed; this implies that hemoglobin absorbs green light more readily than red light.

**Figure 3:**
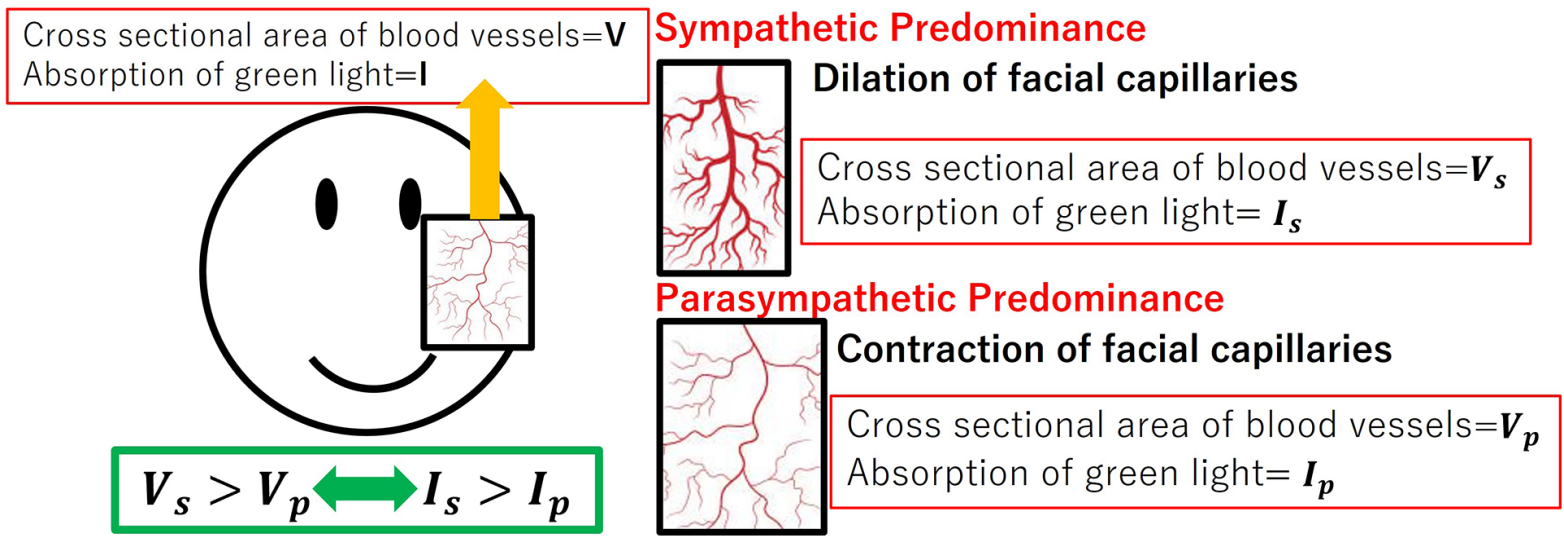
Relationship between capillary dilatation and contraction, as well as the cross-sectional area and absorbance, of the blood vessel.

**Figure 4:**
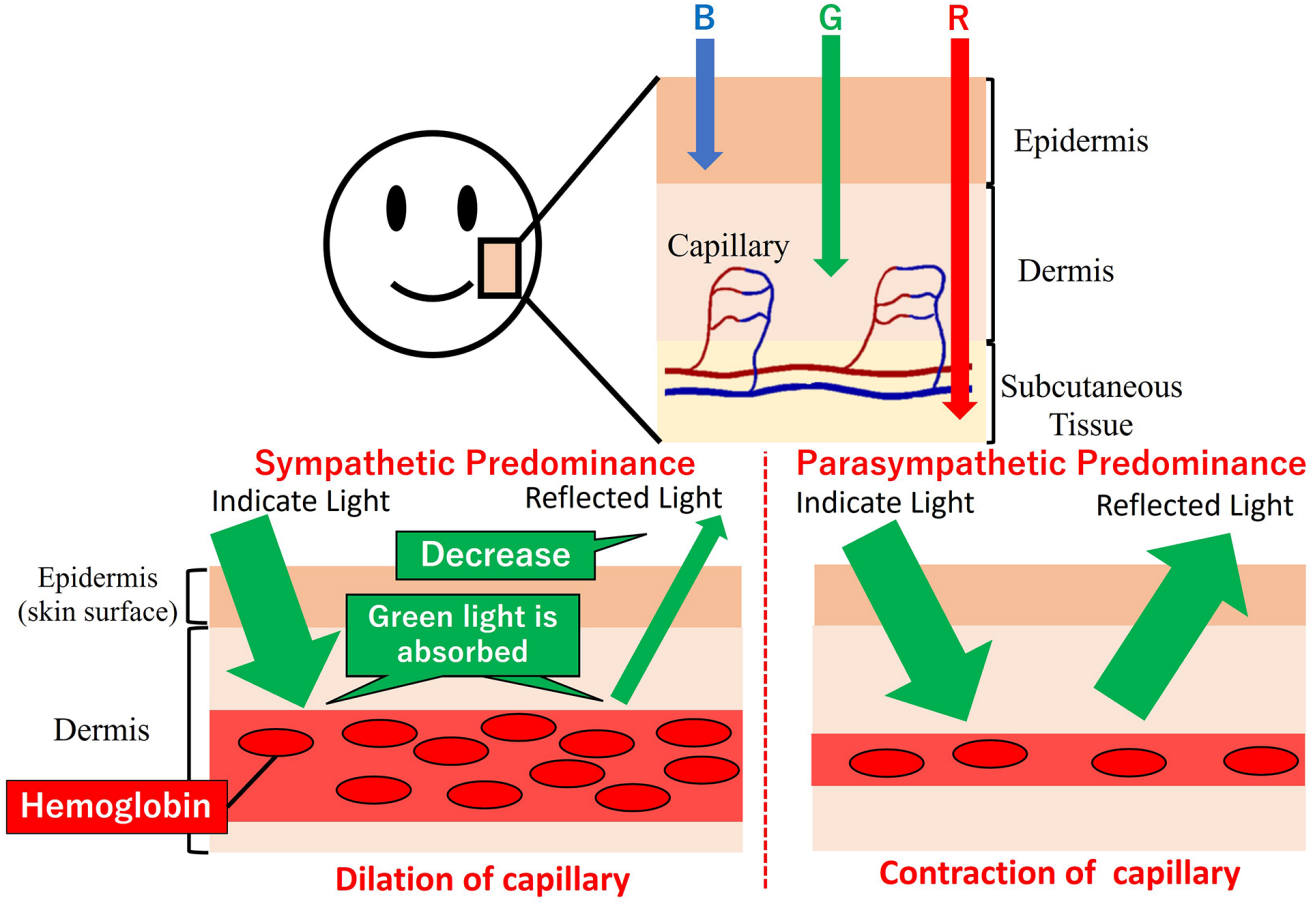
Relationship between autonomic nervous system activity, facial capillaries, and green light.

Green light is readily absorbed by the hemoglobin in the pigmented dermis. So that green light could carry information about the vessels' cross-sectional area. Figure 3 denotes the relationship between capillary dilatation and contraction, as well as the cross-sectional area and absorbance, of the blood vessel. The cross-sectional area of facial capillaries and the amount of green light absorbed are defined as *V* and *I*, respectively. When sympathetic nervous system activity dominates and facial capillaries expand, the cross-sectional area of facial capillaries and the amount of green light absorbed are defined as $V_{s}$ and $I_{s}$. Conversely, when parasympathetic nervous system activity becomes dominant and facial capillaries contract, the cross-sectional area of capillaries and the amount of green light absorbed are defined as $V_{p}$ and $I_{p}$. In this case, $V_{s}>V_{p}$ holds. With increasing cross-sectional area of capillaries, the amount of hemoglobin in the blood and the green light absorbed increases. Therefore, $I_{s}>I_{p}$ holds. Thus, a relationship between the cross-sectional area of capillaries and the amount of green light absorbed exists. Figure 4 shows the relationship between ANSA, facial capillaries, and green light. Light penetrates the human skin as depicted in Figure 1. When the sympathetic nervous system dominates, facial blood vessels dilate (24) because of an increasing fluid volume and the amount of hemoglobin in the capillaries. As shown in Figure 2, the amount of green light reflected from the face decreases because green light is easily absorbed by hemoglobin. Conversely, when the parasympathetic nervous system is activated, facial blood vessel contract. This results in a decrease in fluid volume and the amount of hemoglobin in the capillaries, thereby increasing the amount of green light reflected from the face. Because green light passes through the skin surface, it has information on the skin surface in addition to information on hemoglobin. The information on the skin surface must be reduced because it can become noisy during exercise. Green light penetrates the epidermis while blue light only carries information about the skin's surface. Therefore, by calculating the green-to-blue (G/B) ratio, it is possible to extract vascular information. Table 1 shows the relationship between facial capillaries and green light. The G/B ratio differs from the image pulse wave. Imaging pulse waves measure pulse rate, whereas the G/B ratio extracts information about blood vessel contraction and expansion. Imaging pulse wave technology calculates the pulse from face images (25). However, no technique allows capturing the contraction and expansion of face capillaries. Moreover, the noncontact measurement of the ANSA index is being developed. However, many can be measured only in a stationary state because body movements become noisy (26, 27). In addition, image pulse wave technology is weak against body movement; thus, it cannot be measured without fixing the measurement position. Moreover, processing steps such as image analysis and frequency analysis are necessary. The proposed evaluation method is noncontact and unaffected by the body movement being a very useful for evaluating ANSA. As such, this measurement method can directly evaluate contraction and expansion of facial capillaries and instantaneous ANSA.

**Table 1:** Relationship between autonomic nervous system activity, facial capillaries, and green values.

Item	Sympathetic nervous system activity predominates	Parasympathetic nervous system activity predominates
Facial capillaries	Expansion	Contraction
Blood volume	Increase	Decrease
Amount of hemoglobin	Increase	Decrease
Green value	Decrease	Increase
G/B ratio	Decrease	Increase

### 2.2 Verification experiment

We developed a noncontact evaluation system for ANSA during exercise. To confirm that the system can evaluate the ANSA during exercise, we conducted a verification experiment. To validate the validity of the G/B ratio for the development of a system to detect facial blood vessel information during exercise using a common camera, we conducted an exercise task using an ergometer (indoor cycling IC4, LIFEFITNESS). We chose a cycling exercise using an ergometer because it involves physical movement and exercise intensity is easy to maintain. The experimental subjects were 10 healthy adults (23 ± 1.6 years old). We excluded individuals with a history of upper or lower limb disease, sensitive skin, alcohol allergy, hypertension, diabetes, any preexisting medical condition such as hypertension or diabetes mellitus, fear or anxiety that rendered them unable to perform the exercise task in this study, and any physical condition (e.g., inability to sleep) that rendered them unable to perform the task. We also excluded individuals who were not in good physical condition (e.g., unable to get enough sleep). As we anticipated that makeup could interfere with accurately obtaining changes in face color, we asked those who wearing makeup to remove it before participating in the experiment. This study was conducted in accordance with the ethical principles of the Declaration of Helsinki. All participants provided informed consent, oral and written. This study was approved by the Ethics Committee of Ritsumeikan University (approval number: BKC-LSMH-2022-065).

In this experiment, room brightness posed a challenge as it introduced noise into the experimental data. Room brightness has both long-term effects, influenced by weather and time of day, and short-term effects due to the flow of clouds. Both factors significantly impacted this experiment. Furthermore, the intensity of the circulatory response of the autonomic nervous system varies between the morning and afternoon, necessitating a fixed experiment time. Therefore, to conduct this experiment, it was crucial to create an environment unaffected by changes in room illumination due to weather or day time. To solve these problems, the experiment was conducted in a room completely shielded from outside light. The laboratory was covered with a light-shielding curtain, and the brightness was kept constant in the laboratory using LED lights. The LED light used (HRL-9168, Harvest Japan) emitted white daylight with a luminance of 6,500 K. When the color temperature of the LED is set to a yellowish color such as a light bulb color, it is difficult to evenly cover all the frequency components of visible light. In such an environment, the necessary wavelength of light may be weakened. The brightness of the LED was set to 6,500 K to measure under a white light environment close to natural sunlight. The room dimensions were 4,000 mm × 1,000 mm × 2,000 mm, providing ample room to perform the cycling exercise. The laboratory was bright enough to exercise.

**Figure 5:**
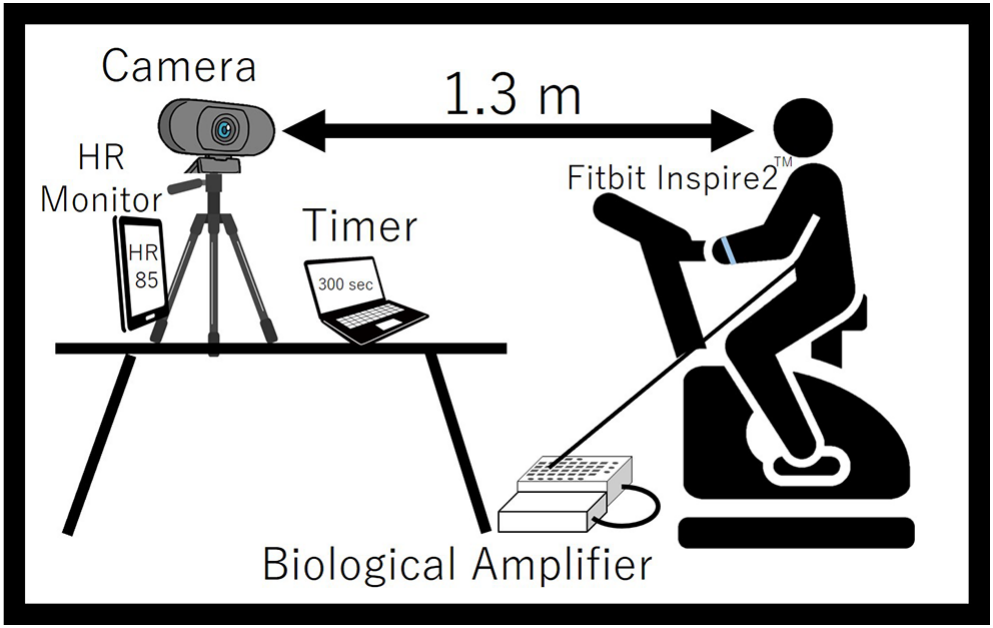
Schematic diagram of the experiment.

A TAWARON-HDC1 camera (TAWARON) was used to capture facial images. The camera was positioned approximately 1.3 m from the participant's head to integrate the pixel values in the images. The white balance and brightness were set to a constant value to keep the shooting conditions constant. The resolution was taken at 1,920 × 1,080. A versatile bio amplifier, PolymateV (Miyuki Giken, Japan), was used to measure electrograms (ECG) at a sampling frequency of 1,000 Hz. The intensity of the participants' exercise during the experiment was self-regulated while observing their real-time heart rate. Therefore, since it was necessary for the experimental subject to check his/her own heart rate during exercise, a wristwatch-type wearable device, $\mathrm{fitbit}\;\mathrm{inspire}2^{\mathrm{TM}}$ (Google, USA), was attached to the participant's arm to allow them to inspect their heart rate during exercise. Skin blood flow is easily influenced by ambient temperature. For human comparison, the optimal conditions are a room temperature of 22°C–26°C and a humidity of 40%–60% (28). In this study, the experiment was conducted under conditions of 22.9°C ± 0.8°C room temperature and 46.0% ± 8.7% humidity. Figure 5 shows a schematic of the experiment.

The experimental procedure comprised an initial 15-min resting period, followed by 15 min of exercise at 40% intensity and 10 min of postexercise rest. No special instructions were given during the exercise task, and the subjects performed cycling exercise freely. The subject's heart rate during the exercise task on the aero bike was considered as the target heart rate and determined using the Karvonen method, see Equation 1 (29).

(1) $$\begin{array}{c} ((220-\mathrm{age})-\mathrm{Resting}\;\mathrm{Rate})\;\times \;\mathrm{Exercise}\;\mathrm{Intensity}\\ \;+\;\mathrm{Resting}\;\mathrm{Rate} \end{array}$$

The experimental subjects performed the exercise task with a wristwatch-type wearable device affixed to their arm to monitor their pulse rate. The device displayed the heart rate in real time, and the subjects were able to exercise at their target heart rate while watching the displayed heart rate.

### 2.3 Analysis methods

**Figure 6:**
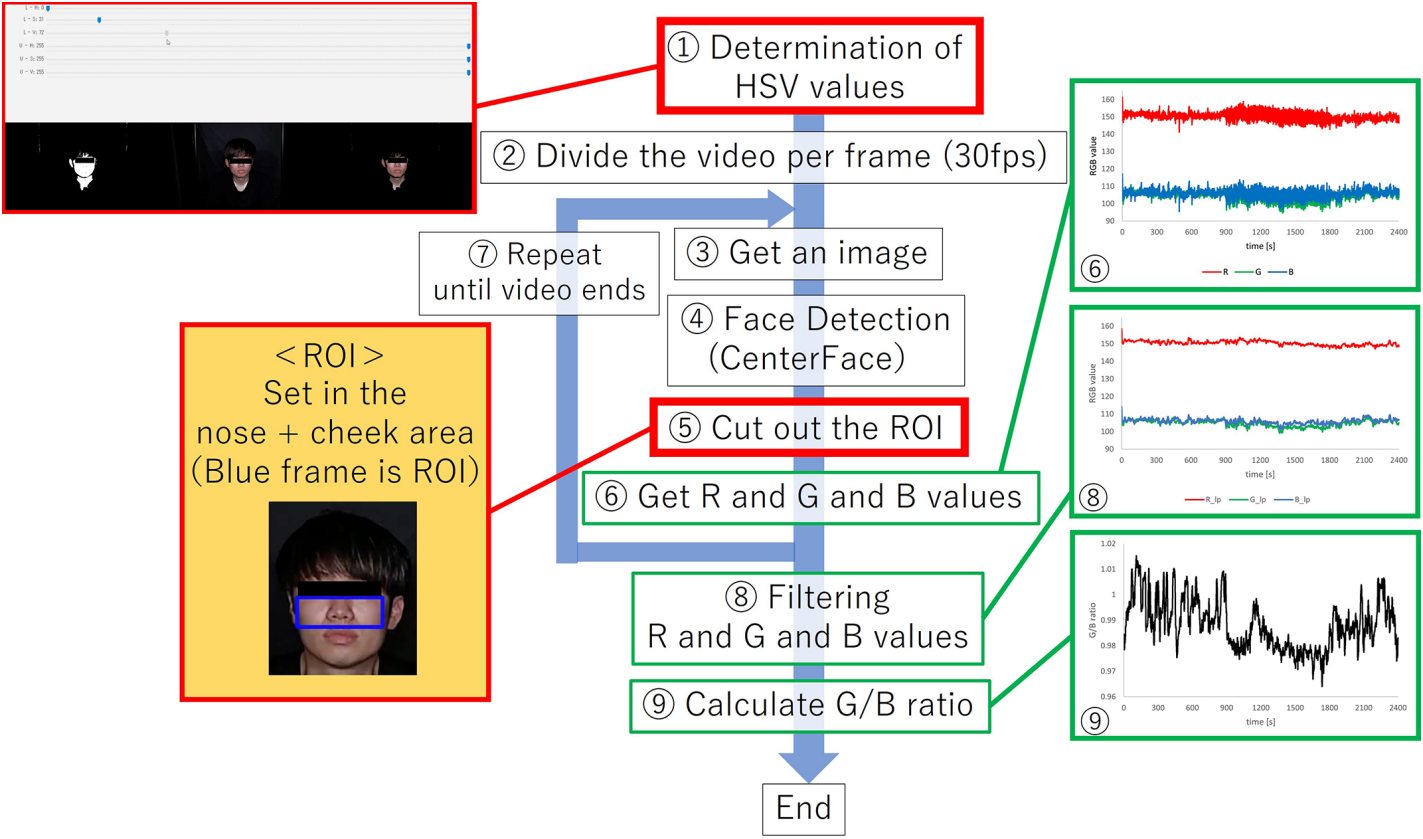
Overview of image processing methods.

A region of interest (ROI) was detected from a face image, to obtain the G/B ratio. Figure 6 shows an overview of the image processing method described below. In Figure 6, processes (1) and (5) surrounded by red lines are processes for eliminating the influence of body motion.

**Figure 7:**
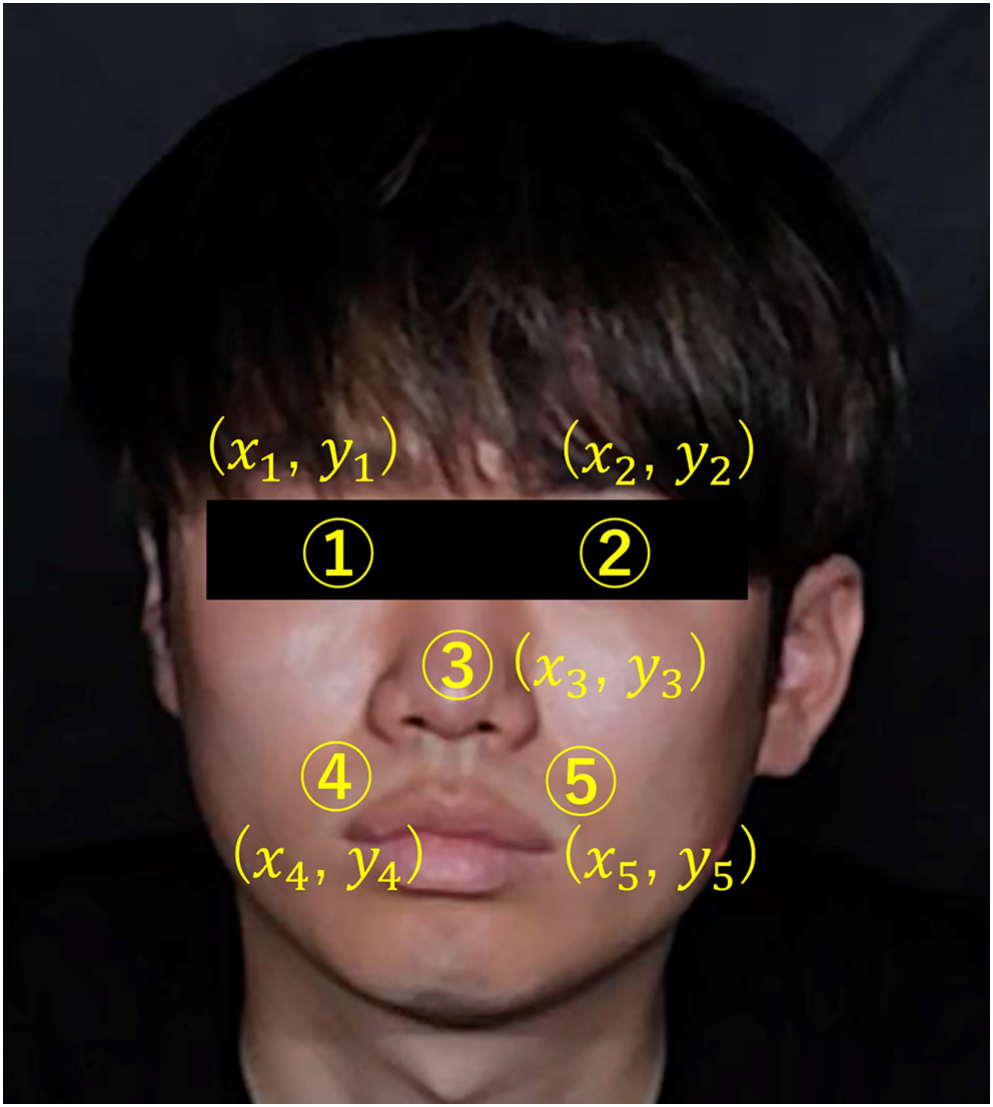
Coordinates of each feature point in the face.

(1) A mask image was created by adjusting the hue saturation value (HSV) range to isolate skin-colored portions. Focusing on these regions, prevented changes in the background due to body movements, ensuring consistent color information acquisition. Upper and lower HSV value limits for creating the mask were set using the software's track bar. It was possible to remove the change of the background color by the body motion by eliminating the color except for the face, and it always succeeded in extracting only the color of the face.
(2) For further image processing, the video was divided into frames. Since the video used was 30 fps and the experiment duration was 40 min, we obtained a total of 72,000 images.
(3) Processing was applied to each image acquired in step (2).
(4) Face detection was performed using the Center Face, a nimble model renowned for swiftly and accurately predicting face box locations and landmarks (30). The Center Face enabled the retrieval of coordinates for each facial feature point, as shown in Figure 7.
(5) ROI images were defined by cropping the images, focusing on the nose and cheeks. The cheek region exhibits a prominent capillary response. However, a ROI limited to the cheeks might be too small to detect capillaries during body movements. To mitigate this, the nose area was included to facilitate measurements unaffected by body movements. By making this range, the capillary information would be able to be measured under the motion. The ROI image was then trimmed into a rectangular shape with specific upper left and lower right coordinates. Here, if the coordinates of the upper left corner of the rectangle are $\left(x_{L},y_{L}\right)$ and the coordinates of the upper right corner are $\left(x_{R},y_{R}\right)$, the coordinates would be calculates as indicated in Equations 2–5. Let us assume that the number of pixels in the ROI image is a * b pixel.
  (2) $$\begin{array}{c} x_{L}\;=\;x_{1}-(x_{2}-x_{1})\;*\;0.5 \end{array}$$
  (3) $$\begin{array}{c} y_{L}\;=\;y_{1} \end{array}$$
  (4) $$\begin{array}{c} x_{R}\;=\;x_{2}\;+\;(x_{2}-x_{1})\;*\;0.5 \end{array}$$
  (5) $$\begin{array}{c} y_{R}\;=\;y_{5}-(x_{3}-x_{5})\;*\;1.7 \end{array}$$
(6) We acquired color information in the ROI area. The average value of red, green, and blue values for each pixel in the ROI region was calculated for each frame. If the matrix of green values in the image is G and the matrix of blue values is B, and the green and blue values at the coordinates (x,y) will be G(x,y) and B(x,y). If H were the matrix of the mask image created after adjusting the HSV in (1), and H(x,y) were the value at the coordinates (x,y). In the matrix H, the skin color is set to 1 and the others to 0. Then, if the matrices of green and blue when only the skin color is extracted are $G_{w}$ and $B_{w}$, and the values at the coordinates (x,y) are $G_{w}(x,y)$ and $B_{w}(x,y)$, the green and blue values at each coordinate are extracted as in Equations 6, 7.
  (6) $$\begin{array}{c} G_{w}(x,y)\;=\;G(x,y)\;*\;H(x,y) \end{array}$$
  (7) $$\begin{array}{c} B_{w}(x,y)\;=\;B(x,y)\;*\;H(x,y) \end{array}$$

Therefore, if the average of green and blue values at this time is $G_{\mathrm{ave}}$ and $B_{\mathrm{ave}}$, the respective values are provided by Equations 8, 9.

(8) $$\begin{array}{c} G_{\mathrm{ave}}\;=\;\frac{\sum_{x\;=\;1}^{a}\;\sum_{y\;=\;1}^{b}\;G_{w}(x,y)}{a\;*\;b} \end{array}$$

(9) $$\begin{array}{c} B_{\mathrm{ave}}\;=\;\frac{\sum_{x\;=\;1}^{a}\;\sum_{y\;=\;1}^{b}\;B_{w}(x,y)}{a\;*\;b} \end{array}$$

(7) To set the ROI area and acquire color information for all frames, steps (3) to (6) were repeated for each frame.
(8) Filtering was applied after acquiring data from the entire video. The obtained RGB values often contained noise from body motions, pulse waves, and respiratory components. To address this, a low-pass filter with a cutoff frequency of 0.1 Hz was used to eliminate noise and extract information regarding vasoconstriction and dilation.
(9) We calculated the G/B ratio by determining the ratio between the green and blue values derived from the RGB values obtained in (8).

ECG data was analyzed using MATLAB software (MathWorks, USA). The first step involved detecting the R wave based on the ECG data measured by the bio amplifier. Next, the RR interval was calculated based on the detected R waves; the RR interval is the interval between the detected R and R waves. Then, the heart rate was derived as reference data for evaluating the G/B ratio. Heart rate variability (HRV) is often used to evaluate ANSA (31, 32). In this study, SDNN and RMSSD were calculated as HRV indices. Clinically, SDNN <50 ms is generally considered to reflect a significant increase in sympathetic activity, and RMSSD <25 ms represents a significant parasympathetic depression (33).

After calculating the G/B ratio and heart rate, we derived time-series data and the mean value of each value. Average values were calculated for 1 min starting 14 min after the beginning of the resting period, 1 min starting 10 min after the beginning of the exercise period, and 1 min starting 1 min after the end of the exercise period. We then derived the changes in each condition using the resting state as a reference. The reason for using the average 10 min after the beginning of exercise as the average during exercise was that all experimental subjects had stable heart rates and other parameters during exercise, and the noise of the G/B ratio was relatively low. Then, heart rate and G/B ratio were expressed as fluctuation values with resting state as 1. One-way analysis of variance (ANOVA) was used for statistical analysis, and Tukey's method was used as a subsequent test. The significance level was set at 0.05. Hypothetically, when sympathetic nervous system activity becomes dominant during exercise, heart rate increases, facial capillaries dilate, and the G/B ratio decreases. However, when parasympathetic nervous system activity dominates postexercise, heart rate decreases, facial capillaries contract, and the G/B ratio increases.

In addition, to investigate the effect of body motion on G/B ratio, the difference between frames was calculated in the video taken during exercise. The total number of pixels between frames calculated as the difference is treated as body motion and evaluated.

## 3 Results

**Figure 8:**
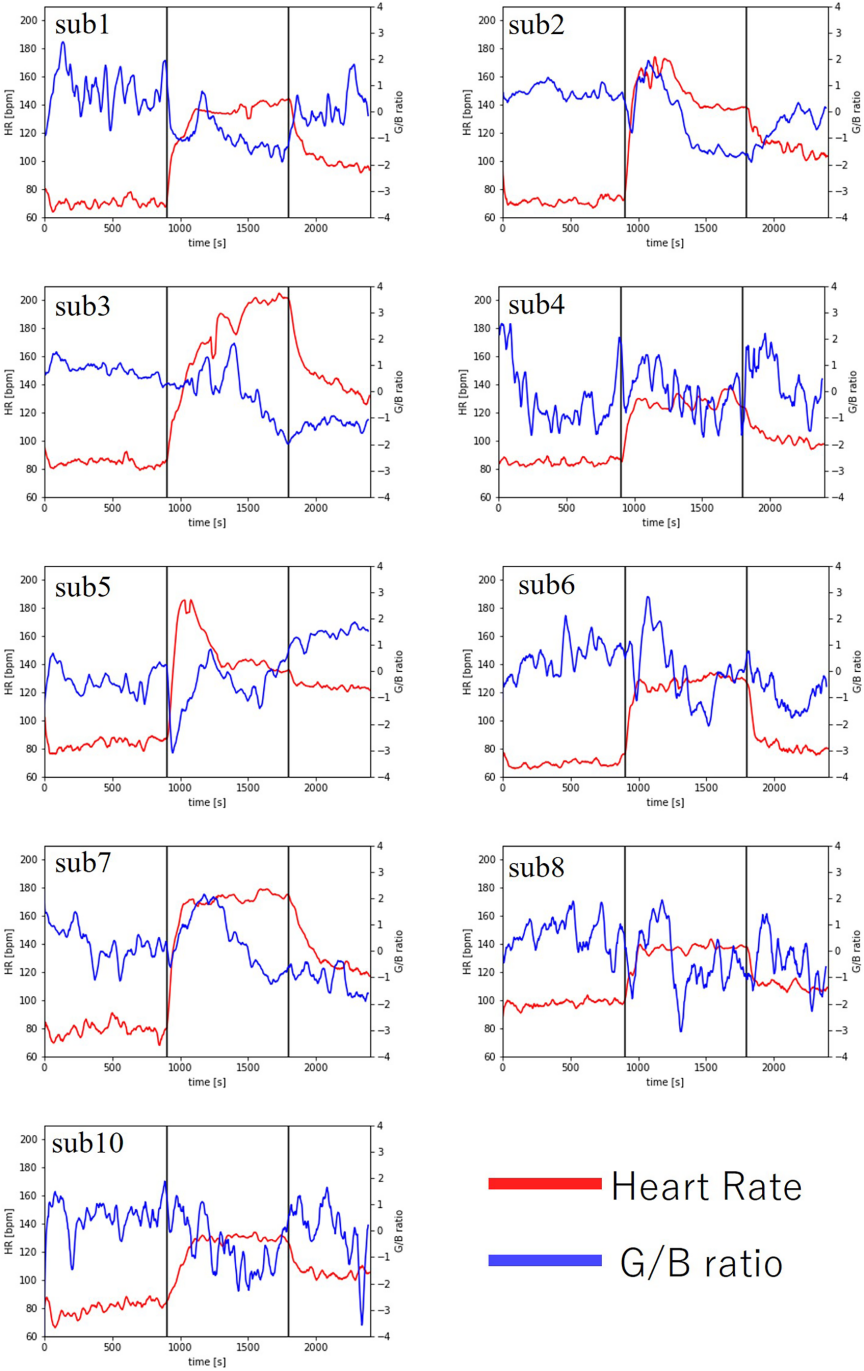
Changes in heart rate and G/B ratio during exercise.

**Figure 9:**
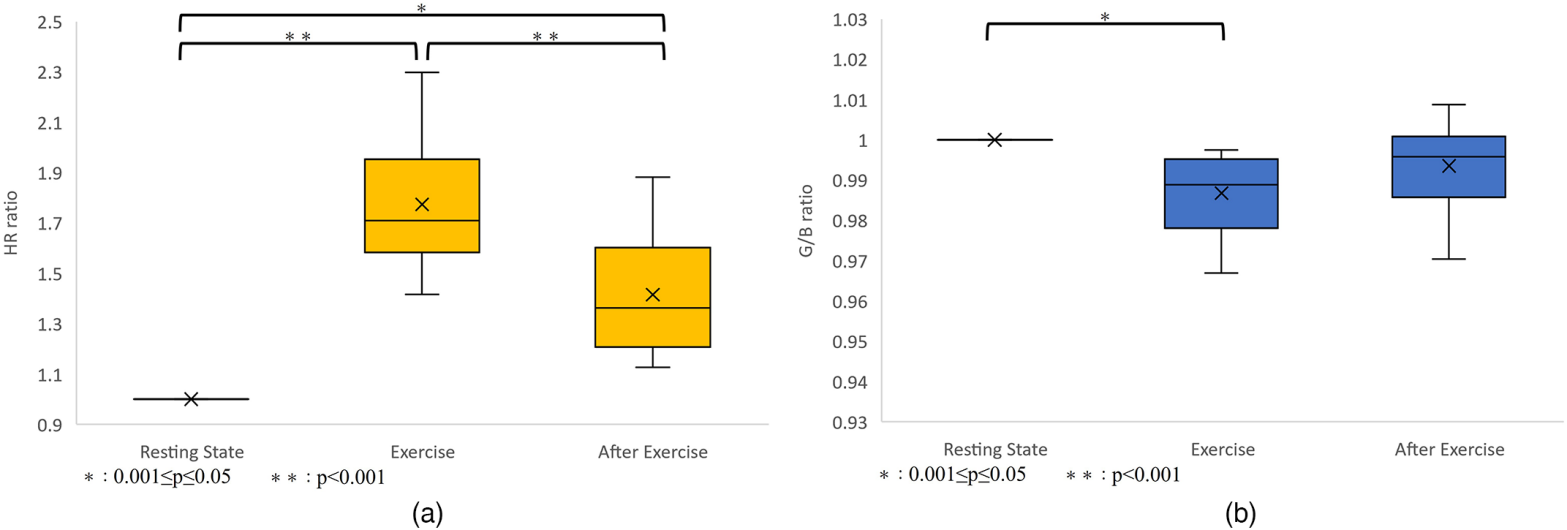
**(a)** Variation in heart rate. *p* < 0.001 between resting-exercise and exercise-after exercise. *p* = 0.001 between resting-after exercise. There were significant differences in all combinations of rest-exercise and exercise-postexercise and rest-postexercise. **(b)** Variation in the G/B ratio. *p* = 0.004 between resting-exercise, *p* = 0.226 between exercise-after exercise, and *p* = 0.175 between resting-after exercise. There was a significant difference only between resting-exercise.

**Figure 10:**
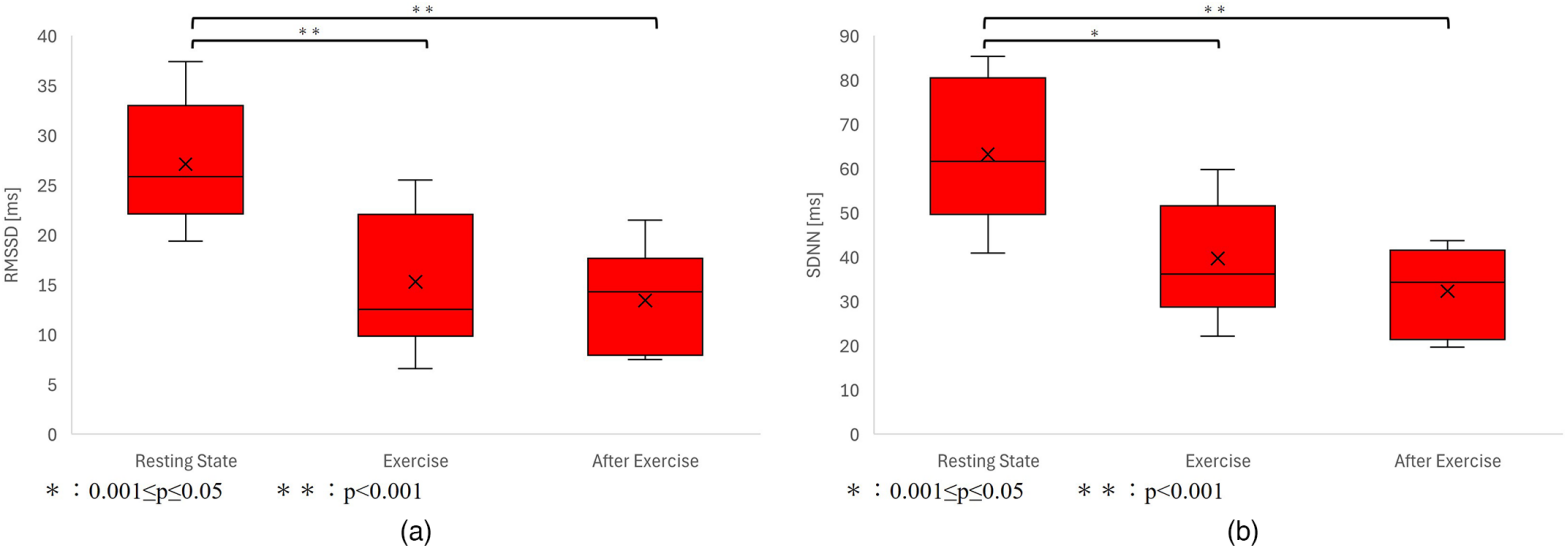
**(a)** Variation in RMSSD. *p* < 0.001 between resting-exercise, *p* = 0.794 between exercise-after exercise, and *p* < 0.001 between resting-after exercise. There was a significant difference only between resting-exercise and resting-after exercise. **(b)** Variation in the G/B ratio. *p* < 0.001 between resting-exercise, *p* = 0.457 between exercise-after exercise, and *p* < 0.001 between resting-after exercise. There was a significant difference only between resting-exercise and resting-after exercise.

As a result, heart rate data and G/B ratio data were obtained from 10 subjects. The results are shown in Figure 8 and Table 2. However, sub9 did not reach the target heart rate during exercise. Therefore, Sub9 was excluded from the analysis. First, Table 2 shows the target heart rates, computed using the subjects' physiological data, including age and resting heart rate. For most subjects, the resting heart rate was 60–80 bpm. However, sub8 showed a high resting heart rate (98 bpm). The subjects then engaged in an exercise task based on their respective target heart rates. Time series of G/B ratio and heart rate data for all subjects are shown in Figure 8. During exercise, the heart rate increased over time, whereas the G/B ratio progressively decreased. Postexercise, the opposite was true. These patterns were observed in most subjects. Additionally, Figure 9 presents box-and-whisker diagrams for G/B ratio and HRV, with the average resting value normalized to 1. Figure 10 shows RMSSD and SDNN for 9 subjects.

**Table 2:** Subject information and target heart rate of each experimental subject.

Number of experimental subjects	Age	Height [m]	Weight [kg]	Resting HR	Target HR
sub1	21	1.66	62	74	124
sub2	23	1.68	55	69	120
sub3	22	1.77	58	70	121
sub4	23	1.86	77	75	124
sub5	22	1.69	60	72	122
sub6	25	1.66	54	74	122
sub7	22	1.7	61	75	124
sub8	24	1.75	58	98	137
sub9	24	1.78	64	75	124
sub10	19	1.74	65	78	127

Our results indicated that the heart rate rapidly increases immediately after the start of exercise decreasing rapidly immediately after the end of exercise. Correspondingly, the G/B ratio decreased with increasing heart rate immediately after the start of exercise and increased when the heart rate decreased immediately after the end of exercise. There was a significant difference in the heart rate at all intervals. There was a significant difference in G/B ratio between resting and exercise phases. RMSSD decreases during exercise, and the mean value for all subjects during exercise is less than 25 ms, indicating that parasympathetic activity is significantly suppressed. The mean value for SDNN for all subjects during exercise is less than 50 ms, indicating that sympathetic activity is significantly increased.

**Figure 11:**
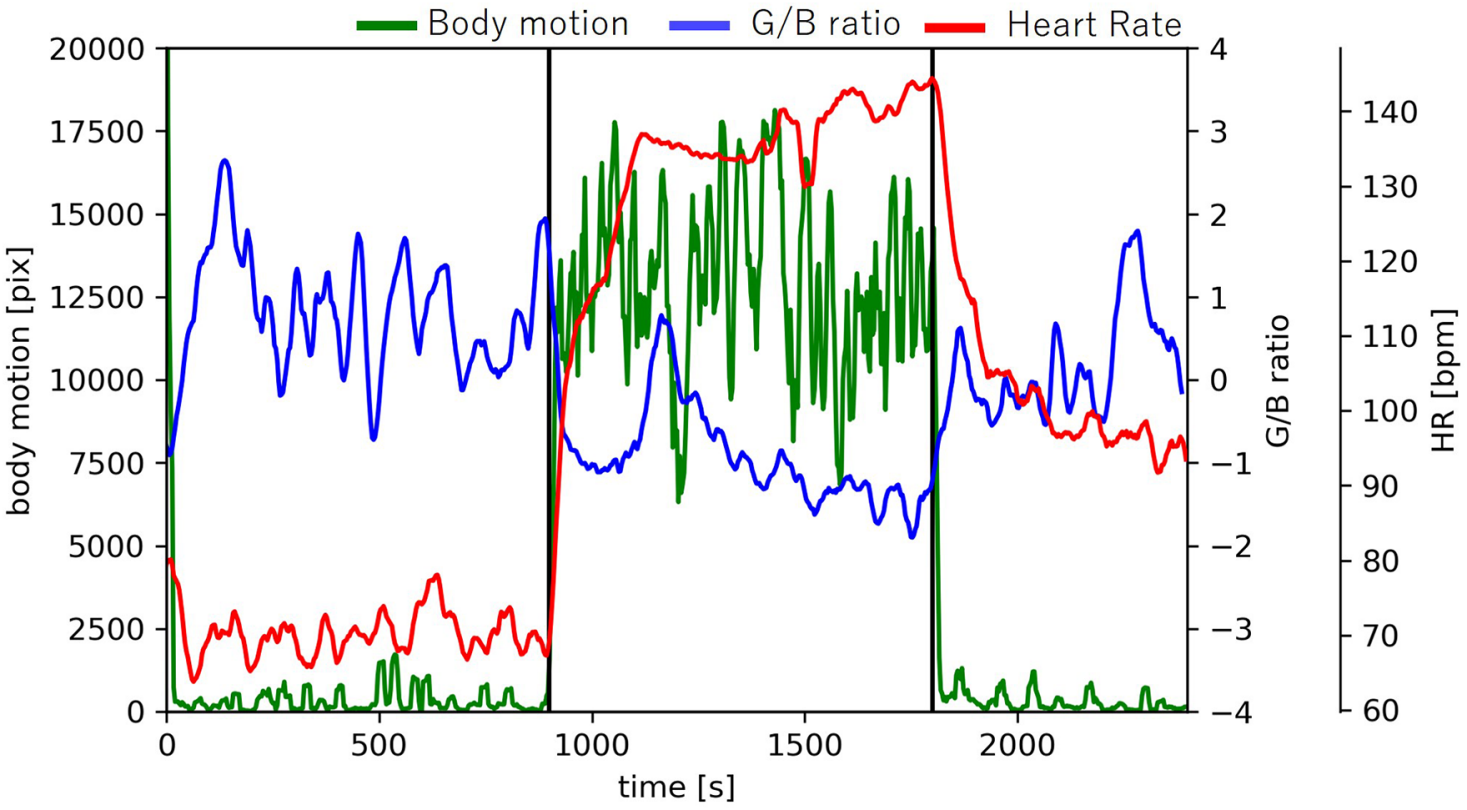
Changes in body motion, G/B ratio, and heart rate.

Figure 11 shows the time-series data of the number of pixels (body movement), G/B ratio and heart rate of sub1. After the start of exercise, body movement increases because the body moves. During exercise, the movement of the body is constant, so the amount of movement is constant. At that time, the G/B ratio decreases, but there was no change in the body movement related to the G/B ratio during exercise.

## 4 Discussion

The results of RMSSD and SDNN show that sympathetic activity increases and parasympathetic activity decreases during exercise. In all subjects, heart rate increased and G/B ratio decreased during exercise. At the beginning of dynamic exercise, the heart rate increases rapidly, primarily because of decreased cardiac vagal regulation (34). With increasing exercise intensity, vagal modulation is further reduced, and heart rate increases due to accompanying sympathetic activation (35). Facial blood vessels dilate with predominating sympathetic activity. The increase in heart rate and decrease in the G/B ratio observed with exercise are considered the result of these ANSA. Therefore, the features of contraction and expansion of the capillary were extracted from the moving image of the face with body movement. To our knowledge, this is the first study to extract features of capillary constriction and dilatation only from a common RGB camera in a noncontact manner during exercise with body movement. The G/B ratio shows features of direct facial capillary constriction and dilatation.

The G/B ratio decreased with increasing heart rate during exercise in all subjects, indicating that the G/B ratio captures vasodilation caused by sympathetic activation. In many subjects, the G/B ratio was lower during the postexercise recovery period than during the preservice period. Conversely, the heart rate was higher during the postexercise recovery period than during the preservice period. These results suggest that the heart rates did not sufficiently recover 10 min after exercise. A previous study on heart rate recovery at different exercise intensities (36) indicated that when the heart rate immediately before the end of exercise was >160, the heart rate at 5 min after the end of exercise was approximately 120 bpm, and at 30 min, it was approximately 100 bpm. For a heart rate of approximately 120 bpm immediately before the end of the exercise, the heart rate 5 min after the end of exercise was approximately 90 bpm, and 30 min after the end of exercise was approximately 85 bpm. In such cases, the heart rate 30 min after the end of exercise was higher than the resting heart rate. These results are consistent with those of our study.

A previous study reported an increase in facial skin blood flow during exercise (37), which aligns with our findings of a decreased G/B ratio during exercise due to vasodilation. Regarding the relationship between heart rate and G/B ratio fluctuations, the G/B ratio also decreased significantly where heart rate increased significantly during exercise, and the G/B ratio fluctuation also tended to decrease where heart rate fluctuation was small. This suggests that the variability in heart rate and capillary dilation/contraction are controlled by the same ANSA.

There are two main mechanisms for autonomic nervous system regulation during exercise: the exercise pressor reflex, in which receptors are stimulated and autonomic nervous system activity is regulated via feedback control, and central command, in which motor commands from higher brain centers directly regulate autonomic nervous system activity (38). Additionally, humans possess a function that allows external changes to alter the internal environment of the body, known as allostasis (39). During exercise, the command for central command, which is a feedforward control, is sent in conjunction with the intention to move, from just before the onset of movement to immediately after its initiation. In other words, by recognizing “exercise,” the brain predicts the upcoming movement and triggers allostasis, thereby issuing central command signals. Since vasodilation and vasoconstriction before and after exercise onset are regulated by central command, changes in the G/B ratio begin responding earlier than body movements (40). In this study, the exercise performed caused a rapid change in heart rate, suggesting a significant influence of central command. Therefore, the change in the G/B ratio is considered reasonable. Also, fluctuations in heart rate and G/B ratio occur almost simultaneously. Vascular expansion and contraction before and after the start of exercise are controlled by the central command (40). Since the G/B ratio may move predictively according to the prediction of the central command, it can be said that there is no significant delay in heart rate. Thus, it can be said that changes in heart rate follow changes in G/B ratio.

Recent advancements in image pulse wave measurement technology have allowed to estimate the heart rate from an RGB camera with high accuracy (41). By taking a moving image of the face only with a camera and combining it with the vascular information measurement system proposed in this study and other high-precision imaging pulse wave measurement techniques, it will be possible to manage physical condition during exercise more accurately in a completely noncontact manner. Heart rate can be used to evaluate optimal exercise intensity, whereas vascular information can be used to assess exercise safety from a cardiovascular perspective. Because the system is entirely noncontact, it eliminates the inconvenience of wearing equipment, allowing for physical condition management in a more natural state and facilitating appropriate exercise evaluation. It is possible to evaluate ANSA only with this system, but by combining it with various indices, it is possible to observe the condition of the body with more detail and accuracy.

The G/B ratio reflects circulatory and thermoregulatory functions. Since there are individual differences in these balance and regulation methods, the variation of the G/B ratio does not perfectly correspond to the variation of heart rate (42, 43). However, the G/B ratio tended to decrease when sympathetic nervous system activity was dominant. In the future, it is necessary to comprehensively evaluate ANSA and to verify whether the G/B ratio is appropriate as an evaluation index of ANSA.

One limitation of this study was the impact of individual differences. For some subjects we had difficulties capturing changes in the G/B ratio due to variations in cardiovascular and autonomic responses, even at the same exercise intensity. Individual differences were also observed in the recovery period. Therefore, it was not possible to eliminate individual differences in response to exercise in this experiment.

In addition, our study was conducted in an environment shielded from external light, which is the ideal experimental environment for the test. Further research is needed to determine whether the same system can be used in environments outside the ideal experimental environment. For practical application, constructing a system that can measure under an environment with rapidly changing external light, such as under sunlight, is necessary. Additionally, constructing an image processing system considering the effect of the change of external light is vital. The camera used in this study had a 30-fps frame rate and a resolution of 1,920 × 1,080. Because our verification experiments were conducted using only a camera with these specifications, it is necessary to examine whether similar experimental results can be obtained using cameras with different specifications.

## 5 Conclusion

Until now, evaluating the ANSA during exercise contactless was difficult. Therefore, we used facial capillaries as an ANSA index and detected contraction and expansion of facial capillaries using only a common RGB camera. We could extract features of contraction and expansion of facial capillaries through image processing during exercise with body movement. There are few restrictions because the measurement method is noncontact. This is an unprecedented evaluation method for ANSA that directly extracts features of contraction and expansion of facial capillaries. Because facial capillaries are controlled by ANSA, they can be considered a new ANSA index. In this study, we proposed a method for measuring capillary vasoconstriction and dilation characteristics during an exercise task using a common camera. Our verification experiment, which involved an exercise task, allowed us to extract these values from all subjects. By combining the camera with other indicators such as heart rate measurements, we could obtain more detailed information about the body function. This enables the sensitive management of physical conditions, potentially leading to healthier lifestyles. This method can be applied in various ways. For instance, it could be used to test heat acclimatization because capillaries dilate with heat acclimation (44). This application will be investigated in future research.

## Data Availability

The raw data supporting the conclusions of this article will be made available by the authors, without undue reservation.

## References

[1] Schneider M Woodworth A Mehrabadi MA. The relationship between exercise habits and stress among individuals with access to internet-connected home fitness equipment: single-group prospective analysis. JMIR Form Res. (2023) 7:e41877. 10.2196/41877 36719817 PMC9947760

[2] Vatovec R Kozinc Z Šarabon N. Exercise interventions to prevent hamstring injuries in athletes: a systematic review and meta-analysis. Eur J Sport Sci. (2019) 20(7):992–1004. 10.1080/17461391.2019.1689300 31680644

[3] Thayer JF Sternberg E. Beyond heart rate variability: vagal regulation of allostatic systems. Ann N Y Acad Sci. (2006) 1088:361–72. 10.1196/annals.1366.014 17192580

[4] Thayer JF Lane RD. A model of neurovisceral integration in emotion regulation and dysregulation. J Affect Disord. (2000) 61(3):201–16. 10.1016/s0165-0327(00)00338-4 11163422

[5] Nieman DC Wentz LM. The compelling link between physical activity and the body’s defense system. J Sport Health Sci. (2019) 8(3):201–17. 10.1016/j.jshs.2018.09.009 31193280 PMC6523821

[6] Hautala AJ Kiviniemi AM Makikallio TH Tiinanen S Seppanen T Huikuri HV Muscle sympathetic nerve activity at rest compared to exercise tolerance. Eur J Appl Physiol. (2008) 102(5):533–8. 10.1007/s00421-007-0618-1 18034260

[7] Pichot V Busso T Roche F Garet M Costes F Duverney D Autonomic adaptations to intensive and overload training periods: a laboratory study. Med Sci Sports Exerc. (2002) 34(10):1660–6. 10.1097/00005768-200210000-00019 12370569

[8] Okano AH Fontes EB Montenegro RA Farinatti PTV Cyrino ES Li LM Brain stimulation modulates the autonomic nervous system, rating of perceived exertion and performance during maximal exercise. Br J Sports Med. (2013) 49(18):1213–8. 10.1136/bjsports-2012-091658 23446641

[9] Périard JD Travers GJS Racinais S Sawka MN. Cardiovascular adaptations supporting human exercise-heat acclimation. Auton Neurosci. (2016) 196:52–62. 10.1016/j.autneu.2016.02.002 26905458

[10] John JH. Guyton and Hall Textbook of Medical Physiology. Amsterdam: Elsevier (2015).

[11] Kellogg DL Jr. In vivo mechanisms of cutaneous vasodilation and vasoconstriction in humans during thermoregulatory challenges. J Appl Physiol. (2006) 100(5):1709–18. 10.1152/japplphysiol.01071.2005 16614368

[12] Schlader ZJ Vargas NT. Regulation of body temperature by autonomic and behavioral thermoeffectors. Exer Sport Sci Rev. (2019) 47(2):116–26. 10.1249/JES.0000000000000180 30632999

[13] Holmes SR King S Stott JRR Clemes S. Facial skin pallor increases during motion sickness. J Psychophysiol. (2002) 16(3):150–7. 10.1027//0269-8803.16.3.150

[14] Zhao C Li GZ Li F Wang Z Liu C. Qualitative and quantitative analysis for facial complexion in traditional Chinese medicine. Biomed Res Int. (2014) 2014:207589. 10.1155/2014/207589 24967342 PMC4054802

[15] Umeda-Kameyama Y Kameyama M Tanaka T Son BK Kojima T Fukasawa M Screening of Alzheimer’s disease by facial complexion using artificial intelligence. Aging. (2021) 13(2):1765–72. 10.18632/aging.202545 33495415 PMC7880359

[16] Steinman J Barszczyk A Sun HS Lee K Feng ZP. Smartphones and video cameras: future methods for blood pressure measurement. Front Digit Health. (2021) 3:770096. 10.3389/fdgth.2021.770096 34870272 PMC8633391

[17] Sanyal S Nundy KK. Algorithms for monitoring heart rate and respiratory rate from the video of a user’s face. IEEE J Transl Eng Health Med. (2018) 6:2700111. 10.1109/JTEHM.2018.2818687 29805920 PMC5957265

[18] Biswal D Mollakazemi MJ Place B Patwardhan A. Heart rate and breathing rate calculated from cheeks and lips using green and derived colors from video. IEEE International Symposium on Smart Electronic Systems (iSES) (Formerly INiS); Chennai, India (2020). p. 23–6

[19] Shokouhmand A Eckstrom S Gholami B Tavassolian N. Camera-augmented non-contact vital sign monitoring in real time. IEEE Sens J. (2022) 22(12):11965–78. 10.1109/JSEN.2022.3172559

[20] Zhang L Fu CH Hong H Xue B Gu X Zhu X Non-contact dual-modality emotion recognition system by CW radar and RGB camera. IEEE Sens J. (2021) 21(20):23198–212. 10.1109/JSEN.2021.3107429

[21] Ortonne JP. Normal and abnormal skin color. Ann Dermatol Venereol. (2012) 139:S125–9. 10.1016/S0151-9638(12)70123-0 23522626

[22] William M. The Structure and Function of Skin, 3rd ed. Cambridge, MA: Academic Press (2012).

[23] Robles FE Chowdhury S Wax A. Assessing hemoglobin concentration using spectroscopic optical coherence tomography for feasibility of tissue diagnostics. Biomed Opt Express. (2010) 1(1):310–7. 10.1364/boe.1.000310/21258468 PMC3005160

[24] Mathias CJ Bannister R. Autonomic Failure: A Textbook of Clinical Disorders of the Autonomic Nervous System. Amsterdam: Oxford University Press (2013).

[25] McDuff DJ. Camera measurement of physiological vital signs. ACM Comput Surv. (2023) 55:1–40. 10.1145/3558518

[26] Takahashi R Ogawa-Ochiai K Tsumura N. Non-contact method of blood pressure estimation using only facial video. Artif Life Robot. (2020) 25:343–50. 10.1007/s10015-020-00622-6

[27] McDuff D Nishidate I Nakano K Haneishi H Aoki Y Tanabe C Non-contact imaging of peripheral hemodynamics during cognitive and psychological stressors. Sci Rep. (2020) 10:10884. 10.1038/s41598-020-67647-6 32616832 PMC7331808

[28] Cui W Cao G Park JH Ouyang Q Zhu Y. Influence of indoor air temperature on human thermal comfort, motivation and performance. Build Environ. (2013) 68:114–22. 10.1016/j.buildenv.2013.06.012

[29] Karvonen MJ Kentala E Mustala O. The effects of training on heart rate; a longitudinal study. Ann Med Exp Biol Fenn. (1957) 35(3):307–15. 13470504

[30] Xu Y Yan W Sun H Yang G Luo J. Centerface: joint face detection and alignment using face as point. Sci Program. (2020) 2020:1–8. 10.1155/2020/7845384

[31] Posada-Quintero HF Dimitrov T Moutran A Park S Chon KH. Analysis of reproducibility of noninvasive measures of sympathetic autonomic control based on electrodermal activity and heart rate variability. IEEE Access. (2019) 7:22523–31. 10.1109/ACCESS.2019.2899485

[32] Mejia-Mejia E Budidha K Abay TY May JM Kyriacou PA. Heart rate variability (HRV) and pulse rate variability (PRV) for the assessment of autonomic responses. Front Physiol. (2020) 11:779. 10.3389/fphys.2020.00779 32792970 PMC7390908

[33] Wang W Shao M Du W Xu Y. Impact of exhaustive exercise on autonomic nervous system activity: insights from HRV analysis. Front Physiol. (2024) 15:1462082. 10.3389/fphys.2024.1462082 39691095 PMC11649657

[34] Freeman JV Dewey FE Hadley DM Myers J Froelicher VF. Autonomic nervous system interaction with the cardiovascular system during exercise. Prog Cardiovasc Dis. (2006) 48(5):342–62. 10.1016/j.pcad.2005.11.003 16627049

[35] Hautala AJ Kiviniemi AM Tulppo MP. Individual responses to aerobic exercise: the role of the autonomic nervous system. Neurosci Biobehav Rev. (2009) 33(2):107–15. 10.1016/j.neubiorev.2008.04.009 18514313

[36] Kaikkonen P Nummela A Rusko H. Heart rate variability dynamics during early recovery after different endurance exercises. Eur J Appl Physiol. (2007) 102(1):79–86. 10.1007/s00421-007-0559-8 17899162

[37] González-Alonso J Crandall CG Johnson JM. The cardiovascular challenge of exercising in the heat. J Physiol. (2008) 586(1):45–53. 10.1113/jphysiol.2007.142158 17855754 PMC2375553

[38] Williamson JW Fadel PJ Mitchell JH. New insights into central cardiovascular control during exercise in humans: a central command update. Exp Physiol.. (2005) 91(1):51–8. 10.1113/expphysiol.2005.032037 16239250

[39] Sterling P Eyer J. Allostasis: a new paradigm to explain arousal pathology. In: Fisher S, Reason J, editors. Handbook of Life Stress, Cognition and Health. Hoboken, NJ: Wiley (1988). p. 629–49.

[40] Ishii K Liang N Oue A Hirasawa A Sato K Sadamoto T Central command contributes to increased blood flow in the noncontracting muscle at the start of one-legged dynamic exercise in humans. J Appl Physiol. (2012) 112(12):1961–74. 10.1152/japplphysiol.00075.2012 22500007

[41] Kurihara K Maeda Y Sugimura D Hamamoto T. Spatio-temporal structure extraction of blood volume pulse using dynamic mode decomposition for heart rate estimation. IEEE Access. (2023) 11:59081–96. 10.1109/ACCESS.2023.3284465

[42] Jacopo ML Gianfranco RMM Salvatore C Giuseppe P Ferdinando I. Investigating feed-forward neural regulation of circulation from analysis of spontaneous arterial pressure and heart rate fluctuations. Circulation. (1999) 99(13):1760–6. 10.1161/01.CIR.99.13.1760 10190888

[43] Romanovsky AA. The thermoregulation system and how it works. Handb Clin Neurol. (2018) 156:3–43. 10.1016/B978-0-444-63912-7.00001-1 30454596

[44] Wendt D Loon LJV Lichtenbelt WD. Thermoregulation during exercise in the heat: strategies for maintaining health and performance. Sports Med. (2007) 37(8):669–82. 10.2165/00007256-200737080-00002 17645370

